# Access to Primary Health Care by older adults from rural areas in Southern Brazil

**DOI:** 10.11606/s1518-8787.2020054002316

**Published:** 2020-12-04

**Authors:** Luiza Santos Ferreira, Laísa Rodrigues Moreira, Simone dos Santos Paludo, Rodrigo Dalke Meucci

**Affiliations:** I Universidade Federal do Rio Grande Faculdade de Medicina Programa de Pós-Graduação em Saúde Pública Rio GrandeRS Brasil Universidade Federal do Rio Grande. Faculdade de Medicina. Programa de Pós-Graduação em Saúde Pública. Rio Grande, RS, Brasil; II Universidade Federal de Pelotas Faculdade de Medicina Programa de Pós-Graduação em Epidemiologia PelotasRS Brasil Universidade Federal de Pelotas. Faculdade de Medicina. Programa de Pós-Graduação em Epidemiologia. Pelotas, RS, Brasil; III Universidade Federal do Rio Grande Instituto de Ciências Humanas e da Informação Rio GrandeRS Brasil Universidade Federal do Rio Grande. Instituto de Ciências Humanas e da Informação. Rio Grande, RS, Brasil; IV Universidade Federal do Rio Grande Faculdade de Medicina Rio GrandeRS Brasil Universidade Federal do Rio Grande. Faculdade de Medicina. Rio Grande, RS, Brasil

**Keywords:** Aged, Health Services Accessibility, Primary Health Care, Family Health Strategy, Rural Health

## Abstract

**OBJECTIVE:**

To characterize the access and use of health services considered reference among the older rural population from a municipality in southern Brazil, whose rural area has full coverage of the Family Health Strategy (FHS), investigating factors associated with the choice of the Basic Family Health Unit (BFHU) as reference.

**METHODS:**

This is a cross-sectional study conducted in 2017 with systematic sampling of rural households in the municipality of Rio Grande (RS) using a standardized in-house questionnaire. We performed descriptive analyses of sociodemographic profile, type of reference service chosen, and reasons for choosing/using the prime-choice service and the nearest BFHU. Poisson regression was used to investigate factors associated with the type of reference service chosen.

**RESULTS:**

Among the 1,030 older adults who participated in the study, 61.4% considered the BFHU a prime choice/reference service mostly due to its proximity (82.6%); the others sought other places due to a greater ease (34.6%) and resoluteness (52.6%). Almost ⅔ of the respondents sought care at the BFHU during the last year, and the reasons differed among those who considered the unit as reference (chronic disease) and those who sought another place (procedures). We also found that the lower the age, income, education, and household-unit distance, the greater the likelihood of the older adult considering the nearest BFHU as reference service.

**CONCLUSIONS:**

The FHS has reached the vulnerable older rural population, approaching an equitable public health system. However, further evaluations are necessary to verify the quality and adequacy of care, given that social structure, enabling factors (such as economic condition), and possible beliefs regarding health still establish the standards for choosing a service.

## INTRODUCTION

Broader concepts and practices have been rebuilding the notion of health care over time. The Alma-Ata^1^ declaration internationally broaden the focus on primary health care (PHC). In Brazil, PHC is being developed by the Family Health Strategy (FHS) – a multidisciplinary work that functions as the gateway to the health care system by the action of teams that assume health responsibility for populations of specific territories^2^. Nowadays, the FHS assists 64.34% of the Brazilian population^6^. Despite the challenges in relocating traditional basic units to the FHS, the access to primary care increased about 450% between 1981 and 2008^7^.

Evaluation is an essential component in the constant expansion and restructuring of health systems, especially regarding the way users access services^5,8-10^. Defining the concept of “access” is a complex task, and no unanimity has been reached among scholars in the field. Its meaning varies according to time and context, but the prevailing one is associated with health services supply capacity^11^.

Evaluating access is even more important when it comes to populations that historically have faced greater barriers to accessing adequate care, such as older rural residents^12^. For presenting greater needs, this group tends to use health services more often^8,15^, so that difficulties in accessing adequate care may entail consequences. Studies comparing the use of primary health care services regarding location found less use and/or worse quality in rural areas than urban areas^4,8,12-16^. In turn, the low variety or quality of locally available services may cause treatment seeking delays or in farther services, resulting in greater physical, emotional, and financial attrition^17^.

Despite the vulnerability of older rural populations and the complexity of developing a care plan, few studies have directed efforts to this portion of the population^4,18^. Given the specific needs and limitations of this group, it is pertinent to investigate how they seek for health services and possible difficulties they encounter. Considering that, this study aims to characterize the access and use of health services considered reference among older adults living in a rural area with full FHS coverage from a municipality in southern Brazil, investigating factors associated with the choice of the Basic Family Health Unit (BFHU) as reference.

## METHODS

This research collected data on the profile of access and use of health services by older residents (individuals aged 60 years or older) of the rural area of Rio Grande (RS). The data represent a cut-off of the cross-sectional study *Saúde da População Rural Rio-Grandina* (Health of the Rio-Grandina Rural Population), conducted by the Graduate Program in Public Health of the Universidade Federal do Rio Grande (FURG). The study investigated health indicators and the pattern of morbidity, access, and use of health services by children up to 5 years old, women of childbearing age, and older residents of the rural area of Rio Grande.

The municipality of Rio Grande is located within the state of Rio Grande do Sul, 350 km from the capital Porto Alegre, and has approximately 200 thousand inhabitants. Of these, 5.5% (about 8,225 individuals) lived in rural areas at the time of the cross-sectional study, and 13.1% among those were older adults^19^. Regarding Public Health Network, 53% of the municipality total area was attended by the FHS, reaching 100% in the rural area. The rural population disposed of 10 family health teams in eight BFHU (one of them functioning as an emergency service, open 24 hours) supported by two Expanded Family Healthcare Centers (EFHC).

Minimum sample size was calculated to estimate the prevalence of the dependent variable (considering 95% confidence level, 70% frequency for estimating results, 3-percentage point margin of error, and 1.5 design effect) and its associated factors (considering 80% power, 10% addition for losses and refusals, and 15% for controlling confounding factors), reaching the number of 857 individuals.

Households within the 23 rural census sectors of the municipality were interviewed between April and November 2017, with a systematic interval of one household for every four sampled, covering approximately 80% of the populations of interest. Households without individuals in the age groups of interest were not considered eligible, but when residents of sampled households fit into the age groups of interest, all eligible individuals were interviewed. Older adults who were institutionalized by the interview period were excluded from the study. Caregivers were interviewed whenever individuals within the age group of interest were physically or mentally incapable of answering the questionnaire.

Data was collected using a standardized questionnaire. Usage profile was analyzed according to the service considered reference by the interviewee through the question: “When you need health care, which is the first service that you go to?”. Response options were posteriorly categorized into “reference BFHU” (the closest unit to participants’ household) or “other service”, including hospitals, private practice or medical offices affiliated to health insurance plans, and services located in other municipalities.

Independent variables were: gender (male and female); age (60 to 69 years; 70 to 79 years; 80 years or older), skin color (white or black/brown/Asian/native American), education level (0 to 4 years; 5 to 8 years; 9 years and over), family income (in tertiles), living alone (no or yes), have a health insurance plan (no or yes), and distance from the nearest BFHU (< 1 km; 1 to 2.9 km; 3 to 5.9 km; 6 to 9.9 km; 10 km or more). The following health status-related variables were also investigated: presenting chronic disease (no or yes) and self-reported health status (good/very good; fair; poor/very poor).

Descriptive statistical analyses of the sample sociodemographic profile, the type of reference service chosen, and the reasons for choosing and using it were performed in the Stata 14.1 statistical software. Poisson regression with robust adjustment for variance was used to investigate factors associated with the type of reference service chosen.

For the adjusted analysis, we developed a conceptual model with hierarchical levels^20^ considering the three categories of factors influencing access to health services^9^ identified by Aday and Andersen^21^: predisposing factors (individual characteristics, prior to the health problem, influencing subject’s propensity to use health services), enabling factors (means that allow care provision), and health needs (related to disease level). Thus, the model employed in the analyses was: 1) distal level: predisposing factors (gender, age, and skin color); 2) intermediate level: enabling factors (education, family income, living alone, having health insurance, and distance from the nearest BFHU); and 3) proximal level: health needs (chronic disease and self-reported health status). The model suffered adjustments for potential confounding factors of the same or higher level, considering p value < 0.20 as the limit to maintain variables in the final model, for controlling positive confusions.

The research project was approved by the Research Ethics Committee of the Universidade Federal do Rio Grande under Opinion No. 51/2017, process 23116.009484/2016-26. All older adults surveyed or their respective caregivers (in case of limitation of the participant) signed the informed consent form.

## RESULTS

Of the 1,351 older adults living in the rural area of Rio Grande, 83.7% (n = 1,131) were sampled for the study, reaching a final number of 1,030 respondents (8.9% of losses and refusals). The sample comprised mostly men, between 60 and 69 years old, white, with 0 to 4 years of education, and living with at least one more person. Only ⅓ of the participants had private health insurance and only 14.9% lived less than one kilometer away from the nearest Basic Family Health Unit (BFHU – Table 1). Median monthly household income was R$1,874. 00 (IQQ = R$1,000.00–R$2,000.00).

**Table 1:** Description of the older rural population sample from the municipality of Rio Grande, Rio Grande do Sul, Brazil, 2017 (n = 1,030).

Variable	n	%
Gender (n = 1,030)		
Male	568	55.15
Female	462	44.85
Age (n = 1,029)		
60 to 69 years	529	51.41
70 to 79 years	327	31.78
80 years or older	173	16.81
Skin color (n = 1,028)		
White	942	91.63
Black/Brown/Asian/Native American	86	8.37
Education (n = 1,017)		
0 to 4 years	703	69.13
5 to 8 years	233	22.91
9 years or more	81	7.96
Live Alone (n = 1,030)		
No	797	77.38
Yes	233	22.62
Household–BFHU distance (n = 842)		
< 1 km	125	14.85
1 km–2,9 km	175	20.78
3 km–4,9 km	101	12.00
5 km–9,9 km	259	30.75
10 km or more	182	21.62
Chronic disease (n = 1,019)		
No	212	20.80
Yes	807	79.20
Health status (n = 1,026)		
Good/very good	590	57.50
Fair	358	34.90
Poor/very poor	78	7.60
Has private health insurance (n = 1,026)		
No	651	63.45
Yes	375	36.55
Reference health service (n = 1,022)		
Reference BFHU (closest to household)	628	61.44
Medical office/health service linked to private health insurance plans	140	13.70
Private practice	94	9.20
Hospital/emergency unit	93	9.10
Other BFHU	48	4.70
Other	19	1.86

BHFU: Basic Family Health Unit.

Regarding seek for health services, almost ⅔ of the respondents (61.4%) considered the BHFU closest to their residence its prime choice/reference service. By stratifying the analyses between the reference BHFU and other care sites, we found different reasons for choosing the service (Table 2). While those who preferred to be attended at their reference BHFU did so mainly due to its proximity from their households, the others underwent follow-up in other services due to habituation, knowing the greater ease and resoluteness of these services in providing the necessary care.

**Table 2:** Reasons for seeking and choosing the reference health service among the older ruralpopulation from the municipality of Rio Grande, Rio Grande do Sul, Brazil, 2017 (n = 1,018).

	Reference service				Total n (%)
	Reference BFHU^a^ (n = 628)		Other service (n = 390)		
	n (%)	Position	n (%)	Position	
Reasons for choosing the reference service*					
Closest to the household	519 (82.6)	1º	25 (6.4)	5º	544 (53.4)
Customary service for providing the necessary treatment	167 (26.6)	2º	205 (52.6)	1º	372 (36.5)
Preference/trust in the service	103 (16.4)	3º	121 (31.0)	3º	224 (22.0)
Easier/faster to get service	58 (9.2)	4º	135 (34.6)	2º	193 (19.0)
Open at the required time	36 (5.7)	5º	44 (11.3)	4º	80 (7.9)
Reference BFHU	17 (2.7)	6º	-	-	17 (1.7)
Difficulty in being attended by the reference BFHU	-	-	07 (1.8)	6º	7 (0.7)
Reasons for seeking the reference service*					
Treatment of chronic disease	295 (47.0)	1º	153 (39.2)	1º	448 (44.0)
Treatment of acute disease	265 (42.2)	2º	148 (38.0)	2º	413 (40.6)
Undergoing clinical procedure	226 (36.0)	3º	19 (4.9)	5º	245 (24.1)
Requesting/undergoing exams	153 (24.4)	4º	52 (13.3)	4º	205 (20.1)
Assessment/routine consultation	112 (17.8)	5º	81 (20.8)	3º	193 (19.0)

BFHU: Basic Family Health Unit

* More than one response per respondent.

As for reasons that led users to seek care, treating already-established diseases (chronic and acute) was the main reason in both groups. Just over half of participants with chronic disease sought care at their reference service (although 79.2% reported presenting with some chronic disease, only 44% sought care).

Tables 3 and 4 outline data on the demand for care at the BHFU closest to older participants’ residences in the 12 months prior to the survey; that is, the BHFU considered as reference unit by the municipal healthcare management. In total, 60.1% of the interviewees sought care at the site, with higher demand among those who considered it as their reference service (75% versus 36%); 87.7% of those evaluated the service positively, with no significant difference among groups.

**Table 3:** Data on demand for care at the reference BFHU in the 12 months prior to the survey for the older rural population from the municipality of Rio Grande, Rio Grande do Sul, Brazil, 2017.

	Reference service		Total n (%)	p
	Reference BFHU N (%)	Other service		
**Sought care at the reference BFHU in the past 12 months (n = 1,014)**	**(n = 626)**	**(n = 388)**		
No	158 (25.2)	247 (63.7)	405 (39.9)	< 0.001
Yes	468 (74.8)	141 (36.3)	609 (60.1)	
**Satisfaction with local service (n = 589)**	**(n = 450)**	**(n = 139)**		0.52
Poor/very poor	7 (1.6)	4 (2.9)	11 (1.9)	
Fair	51 (11.3)	10 (7.2)	61 (10.4)	
Good/very good	392 (87.1)	125 (89.9)	517 (87.7)	

BHFU: Basic Family Health Unit.

**Table 4:** Data on reasons for seeking or not seeking care at the reference BFHU in the 12 months prior to the survey for the older rural population from the municipality of Rio Grande, Rio Grande do Sul, Brazil, 2017.

	Reference service				Total n (%)
	Reference BFHU		Other service		
	n (%)	Position	n (%)	Position	
**Reasons for seeking BFHU in the past 12 months (n = 609)**	(n = 468)*		(n = 141)*		
Treatment of chronic disease	188 (40.2)	1º	37 (26.2)	2º	225 (37.0)
Treatment of acute disease	148 (31.6)	3º	29 (20.6)	3º	177 (29.1)
Undergoing clinical procedure	166 (35.5)	2º	57 (40.4)	1º	223 (36.6)
Requesting/undergoing exams	97 (20.7)	4º	13 (9.2)	4º	110 (18.1)
Assessment/routine consultation	70 (15.0)	5º	6 (4.3)	5º	76 (12.5)
**Reasons for not seeking care at the BFHU in the past 12 months (n = 405)**	(n = 158)*		(n = 247)*		
No need for care	133 (84.2)	1º	87 (35.2)	2º	220 (54.3)
Sought another service	13 (8.2)	2º	104 (42.1)	1º	117 (28.9)
Faced difficulties in receiving care at the BFHU the last time he/she sought care	8 (5.1)	3º	26 (10.5)	3º	34 (8.4)

BFHU: Basic Family Health Unit

* More than one response per respondent.

The reasons that led users to seek care at the respective BHFU differed between participants considering it their prime option and those considering another site as reference: while the former had more general reasons (disease treatment, assessment, examinations), the others sought the BHFU more frequently to undergo clinical procedures.

As for those who did not seek BHFU in the last 12 months (n = 405), the reasons likewise differed between the two groups. Those who considered the BHFU as prime option reported feeling no need to seek some type of care (84.2% versus 35.2%) whereas the others reported preferring another place, which they considered more resolute for obtaining the appropriate care (42.1% versus 8.2%).

Table 5 shows factors associated with choosing the BHFU as reference service, indicating that the lower the age, income, education, and household-unit distance, the greater the likelihood of the older adult considering the nearest BFHU as reference service. Having a private health insurance plan decreased the probability of choosing the BHFU by 25%, and characteristics associated with participants’ health needs were not statistically associated with the choice (Table 5).

**Table 5:** Crude and adjusted analysis of the association between independent variables and choosing the BFHU as reference service for the older rural population from the municipality of Rio Grande, Rio Grande do Sul, Brazil, 2017 (n = 960).

Variable	Crude analysis		Adjusted analysis	
	PR (95%C	p	PR (95%CI)	p
Level 1 – Predisposing Factors				
Gender		0.506		0. 527
Male	1.03 (0.94–1.14)		1.03 (0.94–1.14)	
Female	1		1	
Age		0.026*		0.032*
60 to 69 years	1.17 (1.01–1.35)		1.16 (1.00–1.35)	
70 to 79 years	1.08 (0.92–1.27)		1.08 (0.92–1.27)	
80 years or older	1		1	
Skin color		0.154		0.194
White	1		1	
Black/Brown/Asian/Native American	1.12 (0.96–1.30)		1.13 (0.96–1.34)	
Level 2 – Enabling Factors				
Income in tertiles		< 0.001*		< 0.001*
1st tertile (lower income)	1.43 (1.24–1.64)		1.22 (1.05–1.42)	
2nd tertile	1.34 (1.16–1.55)		1.16 (1.01–1.34)	
3rd tertile (higher income)	1		1	
Education level		< 0.001*		< 0.001*
0 to 4 years	2.25 (1.61–3.61)		1.94 (1.39–2.71)	
5 to 8 years	1.81 (1.27–2.59)		1.57 (1.11–2.22)	
9 years or more	1		1	
Live alone		0.703		0.06
No	1		1	
Yes	0.98 (0.87–1.10)		0.89 (0.78–1.01)	
Household–BFHU distance		< 0.01		0.02
< 1km	1.27 (1.11–1.45)		1.17 (1.03–1.34)	
1 to 4.9 km	1.12 (1.00 – 1.26)		1.10 (0.98–1.23)	
5 km or more	1		1	
Not informed	1.00 (0.86–1.16)		0.95 (0.82–1.10)	
Has health insurance		< 0.001		< 0.001
No	1		1	
Yep	0.70 (0.62–0.79)		0.75 (0.66–0.84)	
Level 3 – Health needs				
Chronic Disease		0.952		0.55
No	1		1	
Yes	1.00 (0.88–1.12)		0.96 (0.86–1.09)	
Health status		0.27*		0.99*
Good/very good	1		1	
Fair	1.06 (0.96–1.18)		1.00 (0.90–1.11)	
Poor/very poor	1.06 (0.88–1.27)		0.99 (0.83–1.20)	

PR: prevalence ratio; 95%CI: 95% confidence interval^;^ BFHU: Basic Family Health Unit.

* linear trend p-value

## DISCUSSION

We found that almost ⅔ (61.4%) of the older residents of the rural area of the municipality of Rio Grande considered the Basic Family Health Unit (BHFU) closest to their residences as a reference health service. The lower the age, education, income, and household-unit distance, the greater the likelihood of the Primary Health Care (PHC) service being chosen as prime option by users. We found a higher percentage of older adults who considered the PHC as a customary service than that reported by another study conducted in Brazil – especially regarding older (36.2%) and rural population (49.2%)^22^.

Reference services have been positively associated with improvements in users’ health condition and system efficiency, since longitudinal monitoring prevents diseases onset and progression, avoiding the overload of emergency services and consultations with specialists for preventable causes^15,22,23^. Moreover, reference services enable the development of a trust relationship between health team and user, strengthening adherence to treatment^2,3, 24^.

Following Aday and Andersen’s categorization^21^, the main health needs that lead older adults to seek the BFHU and other reference services were already-established diseases (chronic and acute), underlining a similar needs profile between both groups. The higher demand for public PHC services may reflect government annual efforts to immunize older adults against influenza through campaigns that, in 2018, exceeded 97% vaccinated individuals and reached 99.39% in 2019^25^.

The literature shows that individuals presenting with chronic diseases have a greater demand for healthcare^5, 9, 23^. However, as already indicated by another study conducted in Brazil^14^, the percentage of service use is still inadequate to users’ needs, given that 40% of participants who reported having some chronic condition did not seek care in the past year.

The groups were quite similar regarding enabling factors, although service supply differed between them. Emergency services and private/affiliated medical offices have a more responsive nature, usually meeting only demands of already sick users; in turn, PHC services aim to develop, besides treatment and rehabilitation, practices of disease prevention, health promotion, and popular participation^2^.

However, these latter purposes did not excel among reasons for seeking for BFHU, which may be explained by the health beliefs and behaviors of the studied population – for example, users and staff unfamiliarity or disengagement regarding PHC broader goals, not deemed as priority^12,23,26^. The high percentage of older adults who reported not having sought chronic illness care, the way they dealt with it, and its possible stigmas may also be implied within such beliefs and behaviors^15,21,24^.

Although the Brazilian Public Health System provides free service for everyone, enabling an easier access to care, respondents with private health insurance or better economic conditions tended to choose other health services as prime option, possibly meaning (as previously identified in the literature) that older adults seek other care sites when they have the means to access more expensive services^17,18, 26^.

Regarding education, the association between lower education and choosing the BHFU as prime option may be related to a greater general self- and health-knowledge, as well as to higher self-medication rates, allowing users to better identify an appropriate service for their health status or decreasing their understanding of the need for follow-up^14,26,27^.

Another enabling factor is health services structure and organization: while distance was decisive for choosing the BFHU as a reference service, those who sought other care sites indicated other elements – such as resoluteness (“offers the treatment I need”), ease and speed in care provision, and confidence in the treatment/professional. Several studies approached the influence of distance to the desired care site, showing that greater distances entail lower service use^3,13,17^, especially among individuals with low mobility, few available service options, and little or no access to means of transportation^4,15,28^. Yet, proximity is not the only factor to guarantee access, as rural residents may be willing to travel longer distances (despite the higher costs and greater physical and emotional attrition) when they assume they would not receive the most suitable care near their residences^10,17,28^.

We found predisposing factors to be only associated with age. Older adults of a younger age may have better mobility and functional capacity, enabling them to actively seek care beyond home visits; but older individuals may require more specific and specialized care, unavailable at primary healthcare level^5,14,29^.

Reasons that lead individuals not to seek service are also important, complementing their perceived organization of the site. Among those who consider the BFHU as the prime care option, the main reason not to seek for it was feeling no need for any type of care, similar to that identified by other studies^5^. However, perceived non-need is a questionable aspect given that our study population demands greater care and, consequently, more frequent qualified assessment of their actual health status. FHS principles are particularly relevant in this context, for it strives for an active search and longitudinal follow-up through family monitoring and home visits conducted by community health agents and technical teams^2,18^.

Being a cross-sectional research and thus susceptible to reversing causality, this research is not intended at investigating cause-effect relationships between the presented variables. This study has some limitations: (1) data related to the service organization was provided only by users, without directly contacting the care services; (2) household-unit distance was also provided by respondents, generating data not as accurate as it would be if collected by georeferencing, as well as a high loss of answers, as some respondents were unable to provide such information.

Given that access is strongly associated with individual perceptions^15^ (i.e., perceived ease of access), the use of other sources to complement data would enrich results. Yet, the exclusive focus on the user is highly relevant and does not invalidate our findings, given that their perceived health needs allow anticipating demands and function as an underlying basis for developing public policies^29^.

As aforementioned, the FHS reached a great amount of the older rural population, especially those in vulnerable situations, approaching an equitable public health system. However, ⅓ of the study group sought other services for follow-up despite having access to BFHU; that is, even with FHS full coverage, social structure, enabling factors (such as economic condition), and beliefs about health still establish the standards for choosing a service.

This finding elucidates the urge for continuous strengthening of the care network for those with greater health needs, as well as spotlighting the profiles of users who did not seek the service. For that, we may develop preventive and health promotion activities targeting individuals who do not perceive the need for follow-up, as well as adapt BFHU structure and organization to attract those who seek another care site. These strategies require new evaluations by managers, researchers, and professionals for expanding knowledge on this matter in different contexts and assessing the adequacy and quality of the service provided.
