# Supplementary material for: Access to Primary Health Care by older adults from rural areas in Southern Brazil
Source: Rev Saude Publica. 2020 Dec 4;54:149. doi: 10.11606/s1518-8787.2020054002316 (PMC7726917; doi:10.11606/s1518-8787.2020054002316)
Supplement: Translation

# Translation: Acesso à Atenção Primária à Saúde por idosos residentes em zona rural no Sul do Brasil

**Authors:** Luiza Santos Ferreira, Laísa Rodrigues Moreira, Simone dos Santos Paludo, Rodrigo Dalke Meucci

**Affiliations:** I Brasil Universidade Federal do Rio Grande. Faculdade de Medicina. Programa de Pós-Graduação em Saúde Pública. Rio Grande, RS, Brasil; II Brasil Universidade Federal de Pelotas. Faculdade de Medicina. Programa de Pós-Graduação em Epidemiologia. Pelotas, RS, Brasil; III Brasil Universidade Federal do Rio Grande. Instituto de Ciências Humanas e da Informação. Rio Grande, RS, Brasil; IV Brasil Universidade Federal do Rio Grande. Faculdade de Medicina. Rio Grande, RS, Brasil

**Keywords:** Idoso, Acesso aos Serviços de Saúde, Atenção Primária à Saúde, Estratégia Saúde da Família, Saúde da População Rural

## Abstract

**OBJETIVO:**

Caracterizar o acesso e utilização de serviços de saúde considerados como referência pela população rural idosa de um município do sul do Brasil, com cobertura de 100% da área rural pela Estratégia Saúde da Família (ESF), investigando os fatores associados à escolha da Unidade Básica de Saúde da Família (UBSF) como serviço de referência.

**MÉTODOS:**

Estudo transversal com amostragem sistemática dos domicílios da área rural do município de Rio Grande (RS), realizado em 2017, através de questionário padronizado, aplicado em domicílio. Foram realizadas análises descritivas referentes a perfil sociodemográfico; tipo de serviço de referência escolhido; e motivos de escolha/utilização do serviço de primeira opção e da UBSF mais próxima. Realizou-se regressão de Poisson para investigar fatores associados ao tipo de serviço de referência escolhido.

**RESULTADOS:**

Participaram do estudo 1.030 idosos, dos quais 61,4% indicaram a unidade básica como serviço de primeira opção/referência. Enquanto aqueles que escolheram ser atendidos na UBSF o faziam principalmente em razão da proximidade (82,6%), os demais procuraram outros locais pela percepção de maior facilidade (34,6%) e resolutividade (52,6%). Quase ⅔ dos entrevistados buscaram atendimento na UBSF no último ano, e os motivos foram diferentes entre aqueles que consideravam a unidade como referência (doença crônica) e aqueles que procuravam outro local (procedimentos). Além disso, quanto menor a idade, renda, escolaridade e distância residência-unidade, maior a probabilidade do idoso considerar a UBSF mais próxima como serviço de referência.

**CONCLUSÃO:**

A ESF tem conseguido atingir a parcela rural idosa em situação de vulnerabilidade, indo ao encontro de um sistema de saúde público equitativo. Todavia, novas avaliações para averiguar adequação e qualidade do atendimento se fazem necessárias, visto que estrutura social, fatores capacitantes (como condição econômica) e possíveis crenças sobre saúde ainda definem o padrão de escolha do serviço.

## INTRODUÇÃO

A atenção em saúde vem se reconstruindo ao longo do tempo, através de conceitos e práticas mais abrangentes. Com a Declaração de Alma-Ata^1^, mais foco e importância foram dados internacionalmente à atenção primária em saúde (APS), que no Brasil está sendo desenvolvida através da Estratégia Saúde da Família (ESF), trabalho multiprofissional, porta de entrada ao sistema de saúde, dirigido a populações de territórios definidos, pelas quais uma equipe assume responsabilidade sanitária^2^. Atualmente, 64,34% da população brasileira é atendida pela ESF^6^. Mesmo com dificuldades no processo de mudança das unidades básicas tradicionais para a ESF, o número de pessoas que acessa a atenção básica aumentou cerca de 450% entre 1981 e 2008^7^.

Na constante ampliação e reestruturação dos sistemas de saúde, a avaliação é essencial, e a forma como se dá o acesso dos usuários aos serviços é um dos aspectos fundamentais desta análise^5,8-10^. O conceito de “acesso” é complexo, e não há unanimidade entre os estudiosos da área. A definição do termo varia conforme tempo e contexto, prevalecendo, porém, um significado associado ao desempenho dos sistemas de saúde quanto a sua capacidade de oferta^11^.

A avaliação do acesso é ainda mais importante quando se trata de populações que tradicionalmente enfrentam dificuldades para obter a devida atenção em saúde, como é o caso da população idosa residente em área rural^12^. Por apresentar maiores necessidades, esse grupo tende a usar mais os serviços de saúde^8,15^, e dificuldades no acesso ao atendimento adequado podem trazer agravos. Estudos que comparam a utilização dos serviços de atenção primária quanto à localização mostram menos uso e/ou pior qualidade na área rural em comparação à urbana^4,8,12^. A baixa variedade ou qualidade dos serviços disponíveis localmente, por sua vez, pode levar à demora na busca por tratamento ou à busca por serviços mais distantes, causando maior desgaste físico, emocional e financeiro^17^.

Apesar da vulnerabilidade da população idosa em área rural e do complexo processo de definição de um plano de cuidado, poucos estudos têm direcionado esforços a essa parcela da população^4,18^. No entanto, uma vez que esse grupo apresenta necessidades e limitações específicas, é pertinente investigar como se dá sua busca por serviços de saúde e possíveis dificuldades encontradas. Assim, o presente estudo objetiva caracterizar o acesso e utilização de serviços de saúde considerados de referência pela população rural idosa de um município do sul do Brasil com cobertura de 100% da área rural pela ESF, investigando fatores de escolha da Unidade Básica de Saúde da Família (UBSF) como serviço de referência.

## MÉTODOS

Esta pesquisa conta com dados referentes ao perfil de acesso e utilização de serviços de saúde por idosos (indivíduos com 60 anos ou mais) residentes na área rural de Rio Grande (RS). Os dados representam um recorte do estudo transversal “Saúde da População Rural Rio-Grandina”, realizado pelo Programa de Pós-Graduação em Saúde Pública da Universidade Federal do Rio Grande (FURG), que investigou indicadores básicos de saúde e o padrão de morbidade, acesso e utilização de serviços de saúde em crianças com até 5 anos de idade incompletos, mulheres em idade fértil e idosos moradores da área rural de Rio Grande.

O município de Rio Grande fica no estado do Rio Grande do Sul, a 350 km da capital Porto Alegre, e conta com aproximadamente 200 mil habitantes. Destes, 5,5% (cerca de 8.225 indivíduos) residiam em área rural no momento do estudo transversal, e dentre os moradores rurais 13,1% eram idosos^19^. Quanto à rede pública de saúde, o município contava com a cobertura de 53% de seu território total pela ESF, chegando a 100% na área rural. Encontravam-se disponíveis à população rural: 10 equipes de saúde da família, instaladas em oito UBSF (uma delas aberta 24 horas, na forma de pronto atendimento) apoiadas por dois Núcleos Ampliados de Saúde da Família (NASF).

Realizou-se cálculo de tamanho de amostra mínimo para estimar a prevalência da variável dependente (considerando nível de confiança de 95%, frequência de 70% para a estimativa do desfecho, margem de erro de 3 pontos percentuais e efeito de delineamento de 1,5) e fatores associados a ela (considerando poder de 80%, acréscimo de 10% para perdas e recusas, e 15% para controle de fatores de confusão), chegando ao número de 857 indivíduos.

Os domicílios dos 23 setores censitários rurais do município foram abordados entre abril e novembro de 2017, com um intervalo sistemático de um domicílio a cada quatro amostrados, totalizando a amostragem de aproximadamente 80% das populações de interesse. Domicílios sem indivíduos nas faixas etárias de interesse não foram considerados elegíveis. Por outro lado, quando os residentes dos domicílios amostrados se enquadravam nas faixas etárias de interesse, todos os indivíduos elegíveis eram entrevistados. Foram excluídos da pesquisa apenas idosos que se encontravam institucionalizados no período de realização das entrevistas. Nos casos de idosos com incapacidade física ou mental para responder o questionário, o cuidador foi entrevistado.

O instrumento utilizado para coleta de dados foi um questionário padronizado. Investigou-se o perfil de utilização de acordo com o serviço considerado como de referência pelo entrevistado, através da pergunta: “Quando o(a) senhor(a) precisa de um atendimento de saúde, qual é o primeiro serviço a que o(a) senhor(a) vai?”. As respostas foram posteriormente categorizadas em “UBSF de referência” (sendo esta a unidade mais próxima da residência do participante) ou “outro serviço”, incluindo hospitais, consultórios médicos particulares e conveniados a plano de saúde privados e serviços localizados em outros municípios.

As variáveis independentes consideradas foram: sexo (masculino; feminino), idade (60 a 69 anos; 70 a 79 anos; 80 anos ou mais), cor da pele (branca ou preta/parda/amarela/indígena), escolaridade (0 a 4 anos; 5 a 8 anos; 9 anos ou mais), renda familiar (em tercis), morar sozinho (não ou sim), possuir plano de saúde (não ou sim) e distância da UBSF mais próxima (< 1 km; 1 a 2,9 km; 3 a 5,9 km; 6 a 9,9 km; 10 km ou mais). Além disso, as seguintes variáveis relacionadas à situação de saúde também foram investigadas: possuir doença crônica (não ou sim) e estado de saúde autorreferido (bom/muito bom; regular; ruim/muito ruim).

Através do programa estatístico Stata 14.1, foram realizadas análises estatísticas descritivas do perfil sociodemográfico da amostra, do tipo de serviço de referência escolhido e dos motivos de escolha e de sua utilização. Além disso, foi realizada regressão de Poisson, com ajuste robusto para variância, para investigar fatores associados ao tipo de serviço de referência escolhido.

Para a análise ajustada, foi construído modelo conceitual hierarquizado por níveis^20^, considerando as três categorias de fatores identificadas por Aday e Andersen^21^ como influenciadoras do acesso aos serviços de saúde^9^: fatores predisponentes (características individuais, prévias ao problema de saúde, que influenciam na propensão do sujeito a utilizar os serviços de saúde), fatores capacitantes (meios que possibilitam a obtenção do atendimento) e necessidades em saúde (relacionadas ao nível de doença). Desse modo, o modelo utilizado nas análises foi: 1) nível distal: fatores predisponentes (sexo, idade e cor da pele); 2) nível intermediário: fatores capacitantes (escolaridade, renda familiar, morar sozinho, possuir plano de saúde e distância da UBSF mais próxima); 3) nível proximal: necessidades em saúde (doença crônica e estado de saúde autorreferido). Foi realizado ajuste para possíveis fatores de confusão do mesmo nível ou superior, considerando o valor p < 0,20 como limite para manter as variáveis no modelo final, a fim de controlar para confusões positivas.

Quanto aos aspectos éticos, o projeto de pesquisa foi aprovado pelo Comitê de Ética em Pesquisa da Universidade Federal do Rio Grande sob o Parecer nº 51/2017, Processo 23116.009484/2016-26. Todos os idosos entrevistados, ou cuidadores (em caso de limitação do participante), assinaram o termo de consentimento livre e esclarecido.

## RESULTADOS

Dos 1.351 idosos moradores da área rural de Rio Grande, 83,7% (n = 1.131) foram amostrados para o estudo, chegando a um número final de 1.030 idosos entrevistados (8,9% de perdas e recusas). A amostra foi formada majoritariamente por homens, entre 60 e 69 anos de idade, cor da pele branca, 0 a 4 anos de escolaridade, e que moravam pelo menos com mais uma pessoa na residência. Somente ⅓ dos participantes tinha plano de saúde privado, e apenas 14,9% dos idosos moravam a menos de um quilômetro de distância da UBSF mais próxima (Tabela 1). A renda domiciliar mensal mediana foi de R$ 1.874,00 (IQQ = R$1.000,00–R$2.000,00).

**Tabela 1:** Descrição da amostra de idosos moradores da área rural do município de Rio Grande, Rio Grande do Sul, Brasil, 2017 (n = 1.030).

Variável	n	%
Sexo (n = 1.030)		
Masculino	568	55,15
Feminino	462	44,85
Idade (n = 1.029)		
60 a 69 anos	529	51,41
70 a 79 anos	327	31,78
80 anos ou mais	173	16,81
Cor da pele (n = 1.028)		
Branca	942	91,63
Preta/Parda/Amarela/Indígena	86	8,37
Escolaridade (n = 1.017)		
0 a 4 anos	703	69,13
5 a 8 anos	233	22,91
9 anos ou mais	81	7,96
Mora sozinho (n = 1.030)		
Não	797	77,38
Sim	233	22,62
Distância domicílio–UBSF (n = 842)		
< 1 km	125	14,85
1 km–2,9 km	175	20,78
3 km–4,9 km	101	12,00
5 km–9,9 km	259	30,75
10 km ou mais	182	21,62
Doença crônica (n = 1.019)		
Não	212	20,80
Sim	807	79,20
Estado de saúde (n = 1.026)		
Bom/muito bom	590	57,50
Regular	358	34,90
Ruim/muito ruim	78	7,60
Possui plano de saúde privado (n = 1.026)		
Não	651	63,45
Sim	375	36,55
Serviço de saúde de referência (n = 1.022)		
UBSF^*^ de referência (mais próxima da residência)	628	61,44
Médico/serviço de saúde vinculado a convênio de saúde privado	140	13,70
Consultório médico particular	94	9,20
Hospital/pronto-socorro	93	9,10
Outra UBSF	48	4,70
Outro	19	1,86

UBSF: Unidade Básica de Saúde da Família.

Quanto à busca por serviços de saúde, quase ⅔ dos entrevistados (61,4%) indicaram a UBSF mais próxima de sua residência como o serviço de primeira opção/de referência. Ao estratificar as análises entre UBSF de referência e demais locais de atendimento, os motivos para escolha do serviço se mostraram diferentes (Tabela 2). Enquanto aqueles que preferiam ser atendidos na sua UBSF de referência o faziam principalmente em função da maior proximidade entre residência e o local de atendimento, os demais seguiam seu acompanhamento em outros serviços por estarem habituados, sabendo que lá conseguiriam o atendimento necessário com maior facilidade e resolutividade.

**Tabela 2:** Motivos para escolha e busca do serviço de saúde de referência por idosos da área rural do município de Rio Grande, Rio Grande do Sul, Brasil, 2017 (n = 1.018).

	Serviço de referência				Total n (%)
	UBSF^a^ de referência (n = 628)		Outro serviço (n = 390)		
	n (%)	Posição	n (%)	Posição	
Motivos para escolha do serviço de referência*					
Mais próximo da residência	519 (82,6)	1º	25 (6,4)	5º	544 (53,4)
Serviço que vou habitualmente, pois oferece o tratamento necessário	167 (26,6)	2º	205 (52,6)	1º	372 (36,5)
Gosto/confio no atendimento	103 (16,4)	3º	121 (31,0)	3º	224 (22,0)
Mais fácil/rápido conseguir atendimento	58 (9,2)	4º	135 (34,6)	2º	193 (19,0)
Fica aberto no horário que preciso	36 (5,7)	5º	44 (11,3)	4º	80 (7,9)
Por ser minha UBSF de referência	17 (2,7)	6º	-	-	17 (1,7)
Dificuldade em ser atendido pela UBSF de referência	-	-	07 (1,8)	6º	7 (0,7)
Motivos para busca do serviço de referência*					
Tratamento de doença crônica	295 (47,0)	1º	153 (39,2)	1º	448 (44,0)
Tratamento de doença aguda	265 (42,2)	2º	148 (38,0)	2º	413 (40,6)
Realização de procedimento	226 (36,0)	3º	19 (4,9)	5º	245 (24,1)
Solicitação/realização de exames	153 (24,4)	4º	52 (13,3)	4º	205 (20,1)
Consulta de revisão/rotina	112 (17,8)	5º	81 (20,8)	3º	193 (19,0)

UBSF: Unidade Básica de Saúde da Família

* Mais de uma resposta por indivíduo.

Quanto aos motivos que levaram os usuários a buscar o serviço, o tratamento de doenças (crônicas e agudas) já instauradas foi a principal causa em ambos os grupos. Cabe destacar que apenas pouco mais da metade daqueles que apresentaram quadro de doença crônica buscaram atendimento em seu serviço de referência (44% buscaram atendimento, enquanto 79,2% relataram apresentar alguma doença crônica).

As Tabelas 3 e 4 descrevem informações referentes à procura de atendimento nos 12 meses anteriores à pesquisa na UBSF mais próxima à moradia dos idosos participantes, isto é, a UBSF considerada como unidade de referência pela gestão municipal em saúde. Dentre os entrevistados, 60,1% buscaram atendimento no local, com procura maior entre aqueles que consideravam o local como seu serviço de referência (75% *versus* 36%). Dentre aqueles que procuraram o serviço, 87,7% avaliaram o atendimento positivamente, não havendo diferença significativa entre os grupos.

**Tabela 3:** Informações relativas à procura por atendimento na UBSF de referência nos 12 meses anteriores à pesquisa por idosos da área rural do município de Rio Grande, Rio Grande do Sul, Brasil, 2017.

	Serviço de referência		Total n (%)	p
	UBSF de referência n (%)	Outro serviço n (%)		
**Buscou atendimento na UBSF de referência nos últimos 12 meses (n = 1.014)**	**(n = 626)**	**(n = 388)**		
Não	158 (25,2)	247 (63,7)	405 (39,9)	< 0,001
Sim	468 (74,8)	141 (36,3)	609 (60,1)	
**Satisfação com o atendimento do local (n = 589)**	**(n = 450)**	**(n = 139)**		0,52
Ruim/muito ruim	7 (1,6)	4 (2,9)	11 (1,9)	
Regular	51 (11,3)	10 (7,2)	61 (10,4)	
Bom/muito bom	392 (87,1)	125 (89,9)	517 (87,7)	

UBSF: Unidade Básica de Saúde da Família.

Os motivos que levaram os usuários a buscar atendimento na respectiva UBSF foram diferentes entre aqueles que consideravam a unidade como sua primeira opção de serviço e aqueles que tinham outro local como referência. Enquanto os primeiros procuravam mais a unidade de forma geral (tratamento de doenças, revisão, exames), os demais buscavam a UBSF mais frequentemente para realização de procedimentos.

Dentre aqueles que não buscaram a UBSF nos últimos 12 meses (n = 405), os motivos para isso se mostraram diferentes entre os dois grupos. Enquanto os que consideravam a UBSF como primeira opção de serviço relataram não perceber necessidade em buscar algum tipo de atendimento (84,2% *versus* 35,2%), os demais relataram como motivo a preferência por outro local, que consideravam mais resolutivo para obter a consulta necessária (42,1% *versus* 8,2%).

A Tabela 5 apresenta os fatores associados à escolha da UBSF como serviço de referência, demonstrando que, quanto menores a idade, renda, escolaridade e distância da residência em relação à unidade, maior a probabilidade do idoso considerar a UBSF mais próxima como serviço de referência. Além disso, possuir plano de saúde privado diminuiu a probabilidade de escolha em 25%, enquanto características relacionadas às necessidades em saúde dos participantes não se mostraram estatisticamente associadas à escolha (Tabela 5).

**Tabela 5:** Análise bruta e ajustada da associação de variáveis independentes com a escolha da UBSF como serviço de referência por idosos da área rural do município de Rio Grande, Rio Grande do Sul, Brasil, 2017 (n = 960).

Variável	Análise bruta		Análise ajustada	
	RP (IC95%)	Valor p	RP (IC95%)	p
Nível 1 – Fatores Predisponentes				
Sexo		0,506		0, 527
Masculino	1,03 (0,94–1,14)		1,03 (0,94–1,14)	
Feminino	1		1	
Idade		0,026*		0,032*
60 a 69 anos	1,17 (1,01–1,35)		1,16 (1,00–1,35)	
70 a 79 anos	1,08 (0,92–1,27)		1,08 (0,92–1,27)	
80 anos ou mais	1		1	
Cor da pele		0,154		0,194
Branca	1		1	
Preta/Parda/Amarela/Indígena	1,12 (0,96–1,30)		1,13 (0,96–1,34)	
Nível 2 – Fatores Capacitantes				
Renda em tercis		< 0,001*		< 0,001*
1º tercil (menor renda)	1,43 (1,24–1,64)		1,22 (1,05–1,42)	
2º tercil	1,34 (1,16–1,55)		1,16 (1,01–1,34)	
3º tercil (maior renda)	1		1	
Escolaridade		< 0,001*		< 0,001*
0 a 4 anos	2,25 (1,61–3,61)		1,94 (1,39–2,71)	
5 a 8 anos	1,81 (1,27–2,59)		1,57 (1,11–2,22)	
9 anos ou mais	1		1	
Mora sozinho		0,703		0,06
Não	1		1	
Sim	0,98 (0,87–1,10)		0,89 (0,78–1,01)	
Distância domicílio–UBSF		< 0,01		0,02
< 1km	1,27 (1,11–1,45)		1,17 (1,03–1,34)	
1 a 4,9 km	1,12 (1,00 – 1,26)		1,10 (0,98–1,23)	
5 km ou mais	1		1	
Não soube informar	1,00 (0,86–1,16)		0,95 (0,82–1,10)	
Possuir plano de saúde		< 0,001		< 0,001
Não	1		1	
Sim	0,70 (0,62–0,79)		0,75 (0,66–0,84)	
Nível 3 – Necessidades em Saúde				
Doença Crônica		0,952		0,55
Não	1		1	
Sim	1,00 (0,88–1,12)		0,96 (0,86–1,09)	
Estado de Saúde		0,27*		0,99*
Bom/Muito bom	1		1	
Regular	1,06 (0,96–1,18)		1,00 (0,90–1,11)	
Ruim/Muito ruim	1,06 (0,88–1,27)		0,99 (0,83–1,20)	

RP: razão de prevalência; IC95%: intervalo de confiança de 95 %; UBSF: Unidade Básica de Saúde da Família.

* Valor p de tendência linear.

## DISCUSSÃO

O presente estudo identificou que quase ⅔ (61,4%) dos idosos moradores da área rural do município de Rio Grande consideraram a UBSF mais próxima da residência como serviço de saúde de referência. Quanto menores a idade, escolaridade, renda e distância da UBSF, maior a probabilidade do serviço de APS ser escolhido como primeira opção pelos usuários. O percentual de idosos que considerou a APS como serviço usual se mostrou elevado quando comparado a outro estudo realizado no Brasil, inclusive no que diz respeito especificamente ao grupo de idosos e população rural (36,2% e 49,2%, respectivamente)^22^.

A definição de um serviço de referência tem se mostrado positivamente associada a melhorias na condição de saúde do usuário e maior eficiência do sistema, uma vez que o acompanhamento longitudinal do indivíduo previne o aparecimento e evolução de doenças, evitando a sobrecarga dos serviços de emergência e de consultas com especialistas por causas evitáveis^15,22,23^. Além disso, tal definição possibilita o desenvolvimento de uma relação de confiança entre equipe de saúde e usuário, fortalecendo a adesão ao tratamento^2,3,24^.

Seguindo a categorização desenvolvida por Aday e Andersen^21^, as principais necessidades em saúde percebidas pelos idosos como motivos para busca tanto das UBSF quanto dos demais serviços de referência foram quadros de doenças já instauradas (doenças crônicas e agudas), mostrando um perfil semelhante de necessidades entre ambos os grupos. A maior procura dos serviços públicos de APS pode ser reflexo de esforços governamentais para anualmente imunizar idosos contra influenza, em campanhas que ultrapassaram a meta de 97% dos idosos vacinados em 2018, chegando a 99,39% de cobertura em 2019^25^.

Além disso, na literatura, ser portador de doença crônica tem se destacado como fator associado a maior procura por atendimento em saúde^5,9,23^. Todavia, como já apontado em outro estudo conduzido no Brasil^14^, o percentual de utilização dos serviços ainda não está adequado às necessidades dos usuários, uma vez que, dentre os participantes que relataram ter algum quadro crônico, 40% não buscaram atendimento no último ano.

Considerando os fatores capacitantes, merece destaque a semelhança entre os grupos, embora o perfil de atendimento dos serviços seja diferente. Enquanto serviços de pronto atendimento e consultórios médicos particulares/conveniados têm caráter mais responsivo, geralmente atendendo apenas a demandas de usuários já enfermos, os serviços de APS têm por objetivo desenvolver, além do tratamento e da reabilitação, práticas de prevenção à doença, promoção da saúde e participação popular^2^.

Essas últimas finalidades, porém, não se sobressaíram entre os motivos para procurar as UBSF, o que pode ser reflexo de crenças e comportamentos em saúde da população estudada, por exemplo por desconhecimento ou desengajamento de usuários e equipe profissional quanto às finalidades mais amplas da APS, não percebidas como prioridade^12,23,26^. O alto percentual de idosos que relataram não ter buscado atendimento para doenças crônicas e a forma como lidaram com a enfermidade e seus possíveis estigmas também podem ser considerados parte destas crenças e comportamentos^15,21,24^.

A oferta gratuita de serviços pelo sistema público de saúde brasileiro permite que todos tenham acesso à atenção mais facilmente. Todavia, ter plano de saúde privado ou melhores condições econômicas mostrou-se associado à escolha de outros serviços de saúde como primeira opção, o que pode significar, como já identificado na literatura, que, tendo meios de acessar serviços mais custosos, os idosos tendem a buscar outros locais de atendimento^17,18,26^.

Quanto à escolaridade, a associação encontrada pode estar relacionada a um maior autoconhecimento geral e em saúde, bem como a taxas mais elevadas de automedicação (permitindo melhor identificação do serviço adequado para o quadro de saúde ou diminuindo a percepção de necessidade de acompanhamento)^14,26,27^.

Outro fator capacitante refere-se à estrutura e organização dos serviços de saúde. Enquanto a distância foi um aspecto decisivo para a escolha das UBSF como serviço de referência, outros elementos – como resolutividade (“oferece o tratamento que necessito”), facilidade e rapidez na obtenção do atendimento e confiança no tratamento/profissional – estiveram presentes nas respostas daqueles que buscam outros locais. A influência da distância até o local de atendimento desejado já foi alvo de várias pesquisas, mostrando que maiores distâncias levam a uma menor utilização dos serviços^3,13,17^, principalmente quando se trata de indivíduos com baixa mobilidade, com poucas opções de serviços disponíveis e pouco ou nenhum acesso a meios de transporte^4,15,28^. Contudo, a proximidade não é o único fator de garantia de acesso, uma vez que moradores de áreas rurais podem estar dispostos a percorrer maiores distâncias quando julgam que não receberiam o atendimento mais adequado às suas necessidades perto de casa, mesmo que o deslocamento signifique maiores custos e maior desgaste físico e emocional^10,17,28^.

Quanto a fatores predisponentes, encontrou-se associação apenas com a idade. Idosos mais jovens podem apresentar melhor mobilidade e capacidade funcional, o que permite a busca ativa por atendimento para além das visitas domiciliares; já indivíduos mais velhos podem requerer atendimentos mais específicos e especializados, indisponíveis no nível primário de atenção^5,14,29^.

Motivos que levam a não buscar um serviço também são aspectos importantes, que complementam a percepção de organização do local por parte do usuário. Entre aqueles que veem a UBSF como primeira opção de atendimento, a principal causa para não a buscar foi considerar que não havia necessidade, realidade semelhante à identificada por outros estudos^5^. Porém, a percepção de não necessidade pode ser questionada, visto se tratar de um grupo populacional que precisa de maiores cuidados e, consequentemente, requer revisões qualificadas mais frequentes para avaliar seu real estado de saúde. Os princípios da ESF se mostram bastante pertinentes neste contexto, uma vez que esta prima pela busca ativa e acompanhamento longitudinal através de acompanhamento familiar e visitas domiciliares feitas por agentes comunitários de saúde e equipe técnica^2,18^.

Deve-se considerar que, como pesquisa transversal, o presente estudo não intenciona investigar relações de causa e efeito entre as variáveis apresentadas, visto que o delineamento utilizado está suscetível à causalidade reversa. Além disso, cabe ressaltar como limitações: (1) características referentes à organização do serviço foram coletadas apenas através dos usuários, não tendo ocorrido contato direto com os serviços de atendimento; (2) as medidas referentes à distância entre residência e UBSF também foram coletadas através do relato dos entrevistados, gerando dados não tão precisos quanto os georreferenciados e perda elevada de respostas, visto que alguns idosos não tinham essa informação.

Considerando que o acesso está fortemente relacionado às percepções individuais^15^ (isto é, quão fácil o usuário percebe ser o acesso), a complementação dos dados por diferentes fontes enriqueceria os resultados. Ainda assim, o foco exclusivo no usuário é de grande relevância e não inviabiliza os achados expostos, uma vez que as necessidades em saúde percebidas pelo usuário permitem antecipar demandas e são uma das bases para elaborar políticas públicas^29^.

Por fim, conforme evidenciado, a ESF tem conseguido alcançar grande parte da população rural idosa, principalmente grupos em situação de vulnerabilidade, indo ao encontro de um sistema de saúde público equitativo. Todavia, apesar de ter acesso às UBSF, ⅓ do grupo estudado busca outros serviços para acompanhamento. Ou seja, mesmo com uma cobertura de 100% pela ESF, estrutura social, fatores capacitantes (como condição econômica) e crenças sobre saúde ainda definem o padrão de escolha do serviço a ser procurado.

Desta forma, destaca-se a necessidade de continuar a fortalecer a rede de cuidado daqueles que apresentam maiores necessidades em saúde, direcionando a atenção aos perfis de usuários que não buscaram o serviço. Pode-se desenvolver atividades de caráter preventivo e de promoção à saúde direcionadas àqueles que não percebem necessidade de acompanhamento, bem como adequar a estrutura e a organização para trazer à UBSF os que buscam outro local de atendimento. Para isso, novas avaliações por parte de gestores, pesquisadores e profissionais são pertinentes, a fim de ampliar os conhecimentos sobre o tema em diferentes contextos e averiguar a adequação e a qualidade do atendimento oferecido.

**Tabela 4:** Informações relativas aos motivos de busca e não busca por atendimento na UBSF de referência nos 12 meses anteriores à pesquisa por idosos da área rural do município de Rio Grande, Rio Grande do Sul, Brasil, 2017.

	Serviço de referência				Total n (%)
	UBSF de referência		Outro serviço		
	n (%)	Posição	n (%)	Posição	
**Motivos para busca da UBSF nos últimos 12 meses (n = 609)**	(n = 468)*		(n = 141)*		
Tratamento de doença crônica	188 (40,2)	1º	37 (26,2)	2º	225 (37,0)
Tratamento de doença aguda	148 (31,6)	3º	29 (20,6)	3º	177 (29,1)
Realização de procedimento	166 (35,5)	2º	57 (40,4)	1º	223 (36,6)
Solicitação/realização de exames	97 (20,7)	4º	13 (9,2)	4º	110 (18,1)
Consulta de revisão/rotina	70 (15,0)	5º	6 (4,3)	5º	76 (12,5)
**Motivos para a não busca de atendimento na UBSF nos últimos 12 meses (n = 405)**	(n = 158)*		(n = 247)*		
Não precisou de atendimento	133 (84,2)	1º	87 (35,2)	2º	220 (54,3)
Buscou atendimento em outro serviço	13 (8,2)	2º	104 (42,1)	1º	117 (28,9)
Encontrou dificuldade em obter atendimento na UBSF na última vez que buscou	8 (5,1)	3º	26 (10,5)	3º	34 (8,4)

UBSF: Unidade Básica de Saúde da Família

* Mais de uma resposta por indivíduo.
